# Pumping iron: a potential target for novel therapeutics against schistosomes

**DOI:** 10.1016/j.pt.2007.08.018

**Published:** 2007-12

**Authors:** Amber Glanfield, Donald P. McManus, Greg J. Anderson, Malcolm K. Jones

**Affiliations:** 1 Division of Infectious Diseases and Immunology, The Queensland Institute of Medical Research, 300 Herston Road, Herston, Qld 4006, Australia; 2 Division of Population Studies and Human Genetics, The Queensland Institute of Medical Research, 300 Herston Road, Herston, Qld 4006, Australia; 3 School of Population Health, The University of Queensland, Public Health Building, Herston, Qld 4072, Australia; 4 School of Veterinary Sciences, The University of Queensland, St Lucia, Qld 4072, Australia

## Abstract

Parasites, as with the vast majority of organisms, are dependent on iron. Pathogens must compete directly with the host for this essential trace metal, which is sequestered within host proteins and inorganic chelates. Not surprisingly, pathogenic prokaryotes and eukaryotic parasites have diverse adaptations to exploit host iron resources. How pathogenic bacteria scavenge host iron is well characterized and is reasonably well known for a few parasitic protozoa, but is poorly understood for metazoan parasites. Strategies of iron acquisition by schistosomes are examined here, with emphasis on possible mechanisms of iron absorption from host serum iron transporters or from digested haem. Elucidation of these metabolic mechanisms could lead to the development of new interventions for the control of schistosomiasis and other helminth diseases.

## Filling the gaps in metabolic pathways

The recent release of the genome sequence information of a range of parasites has provided a plethora of tools for the study of parasite biology [1]. Despite these advances, Scholl and colleagues [2] state that, for the malaria genome at least, there remain many ‘gene gaps’ where little is known of gene control of specific metabolic or development mechanisms. One field of relative ignorance is how parasites acquire essential trace elements, in particular iron, from their host environment [2]. The gene gaps for malaria become veritable chasms for less intensively studied metazoan parasites, such as schistosomes. Although schistosomes are known to have strong dependence on trace metals, little is known of the acquisition biology of the elements. Adult schistosomes live in and feed on the iron-rich environment of host blood [3,4]. However, newly penetrant schistosomules absorb iron before their gut is differentiated [4], implicating the parasite surface in iron acquisition, as occurs with many other small molecular weight serum components [5]. It is probable that iron acquisition from the host environment constitutes a crucial factor in parasite survival, which has the potential to be exploited by therapeutics.

## Iron: an element essential for life

Iron contributes ∼5% of the Earth's crust, and is a trace requirement for virtually all prokaryotic and eukaryotic organisms [6]. The element readily and reversibly transitions between two oxidation states, Fe^2+^ (ferrous) and Fe^3+^ (ferric). This property has enabled eukaryotes and prokaryotes to use iron for many crucial biological reactions [6]. Iron forms the active centre of numerous diverse proteins, including ribonucleotide reductase, mitochondrial aconitase and haem-containing proteins, such as the cytochromes and iron-sulfur (Fe-S) proteins of the electron transport chain [7]. Iron confers oxygen-binding ability to haem moieties of haemoglobin (Hb) and myoglobin; iron-containing proteins are central to the metabolism of collagen, tyrosine and catecholamines [7–9], and for innate and acquired immunological responses in mammals. Despite its essential role in diverse reactions, iron, if not appropriately chelated, can convert oxygen to toxic free radical species by the iron-catalyzed Haber-Weiss and Fenton reactions. These reactive oxygen radicals are able to attack membrane lipids, proteins and DNA [6,8]. Iron, therefore, presents a dilemma for living systems – although essential for life, it is also harmful. This paradox has led to the evolution of sophisticated mechanisms for regulating the absorption, transport and storage of cellular iron [7]. Iron homeostasis is most tightly regulated at uptake [8]. There are extensive data on iron uptake, transport and regulation in prokaryotes, plants, yeast and vertebrates. By contrast, the mechanisms of iron accumulation in parasites, particularly parasitic helminths, are neglected.

## Iron: a limiting factor in pathogen invasion

Tight regulation of iron in mammalian hosts presents an obstacle to invading pathogens, which also require this essential element. Hosts bind iron in proteins or inorganic chelates, and present an iron-restricted environment to pathogenic bacteria and parasites. Indeed, hosts ‘withhold’ iron as an integral strategy of innate immunity [10] and host–pathogen competition for the element is a deciding factor in the success of infection [10,11]. Microbial pathogens, consequently, have evolved efficient mechanisms to exploit host iron sources. Most iron circulating in mammalian blood is either in the form of haem (bound in haemoglobin within erythrocytes) or reversibly bound to glycoprotein carriers, such as transferrin (Tf). In addition to these primary resources, invading pathogens use a diverse range of host molecules for iron [12] (Box 1).

## Iron uptake in pathogenic prokaryotes

Strategies for iron acquisition from hosts have been studied extensively for prokaryotic pathogens [12]. Uptake mechanisms include the synthesis of siderophores to bind Fe^3+^, and production of specific ligands to entrap and strip host iron carriers (Table 1). One iron-entrapment strategy commonly employed involves the targeted use of proteases and reductases to cleave and reduce bound iron to free Fe^2+^ for internalization by a range of transporters [12]. Pathogenic members of the *Pasteurellaceae* and *Neisseriaceae* acquire iron directly from host transferrin by means of specific receptor-mediated uptake [13–15]. Prokaryote iron uptake is regulated post-transcriptionally in response to iron availability. Usually, iron transporters are detectable only when the bacteria are under iron-restricted conditions [12,14].

## Iron uptake in parasitic Protozoa

Iron-uptake strategies of parasitic protozoans are summarized in Table 2. As is also observed for some pathogenic bacteria, the intracellular location of some protozoans presents those species with major obstacles of iron restriction that the parasites must overcome [16]. Iron is essential for growth of *Leishmania*, *Plasmodium*, *Trichomonas* and *Trypanosoma* species *in vitro*, and this development can be disrupted by administration of iron chelators [17]. Although transferrin receptors have been preliminarily identified for all these protozoan genera, the former two have not held up in further investigations [17,18]. Molecular characterization of a transferrin receptor exists only for *Trypanosoma* [19].

For intracellular parasites, such as *Leishmania* and *Plasmodium*, iron uptake might be mediated through breakdown of haem or by ferrous iron uptake of cytosolic iron (Table 2). It is known that incubation of *Leishmania chagasi* in the presence of bathophenanthroline, which chelates Fe^2+^ but not Fe^3+^, inhibits iron uptake. *Leishmania* probably cleaves iron from host transferrin using a ferric reductase, and internalizes this iron via a ferrous iron transporter [18]. In support of this hypothesis, it has been recently shown that *Leishmania amazonesis* amastigotes express a Fe^2+^ iron transporter 1 (LIT1) [20]. LIT1 promoted iron transport in LIT1 null amastigotes and endogenous LIT1 was upregulated in normal amastigotes cultured in iron-deprived media. Furthermore, LIT1-deficient amastigotes were unable to replicate in macrophages and were avirulent in mice [20]. Trichomonads grown in iron-deficient media also lose virulence [21], indicating that iron transporters are important virulence factors for these flagellates.

## Iron uptake in metazoans

Apart from the well characterized iron metabolism pathways of mammals [7,9,22], knowledge of metazoan iron homeostasis is limited. Some data exist for the iron-related proteins of insects [23], but these data are mostly related to the biology of transferrins and ferritins, and not molecules for iron uptake [24]. An emerging field is in the understanding of haem acquisition and breakdown mechanisms of haematophagous metazoans. Many blood-feeding insects engorge on blood, and the abundance of reactive haem is problematic. The triatomine hemipteran, *Rhodnius prolixus*, for example, processes haem, not by the classical pathways resulting in biliverdin (BV) IX, carbon monoxide (CO) and iron, but by unique reactions resulting in dicysteinyl-BV IXgamma, CO and iron [25]. Some of the haem is absorbed by the parasite and can be catabolized. Interestingly, iron produced from haem degradation is stored, in the presence of ferritin, in mid-gut and pericardial cells of the insect [25]. These findings raise the possibility that haem is a major source of iron in haematophagous metazoans. However, for helminths, this hypothesis requires the presence of haem oxygenases (HO) capable of liberating iron from haem, which have yet to be identified in worm genomes [26].

Roundworms and flatworms possess haem-containing proteins, but are said to lack the biosynthetic machinery for haem [26]. These data arise from biochemical assays of haem-synthesis in a range of free-living and parasitic helminths, including *Schistosoma mansoni*. Rao and colleagues [26] suggest that helminths scavenge haem from dietary or environmental sources. In the case of schistosomes, the gastrodermal lumen represents a major source of haem, most of which is sequestered in haematin [27]. It is noteworthy that sequences for known enzymes of haem biosynthesis, such as δ–aminolevulinic acid dehydratase (ALAD) and porphobilinogen deaminase (PBGD), have been reported for the schistosome expressed sequence tag (EST) datasets [28,29], suggesting that these helminths, at least, have a capacity to make haem. Whether the source of haem for haematophagous helminths is by *de novo* synthesis or salvage is an unresolved and intriguing question. It also remains to be determined if helminths can salvage iron from haem through action of HO.

## Iron and schistosomes

There is solid evidence that iron is used by schistosomes for development and reproduction. Schistosomes sequester iron in the gastrodermal lumen [3], held largely in haem and haematin [27]. In addition, extensive iron is stored within isoforms of the highly conserved iron-storage protein, ferritin (Fer). One isoform, Fer-2, is typical of somatic tissues, the other, Fer-1, occurs as an abundant component of yolk platelets of vitelline cells (eggshell precursors and possible yolk cells) [30,31]. Female schistosomes express 15-fold more Fer-1 than males, but equal amounts of Fer-2 occur in both sexes [31,32]. Roles for this abundant egg-associated iron store include early embryogenesis [31] and stabilization of cross-linked proteins in eggshell formation [32].

**Figure 1:**
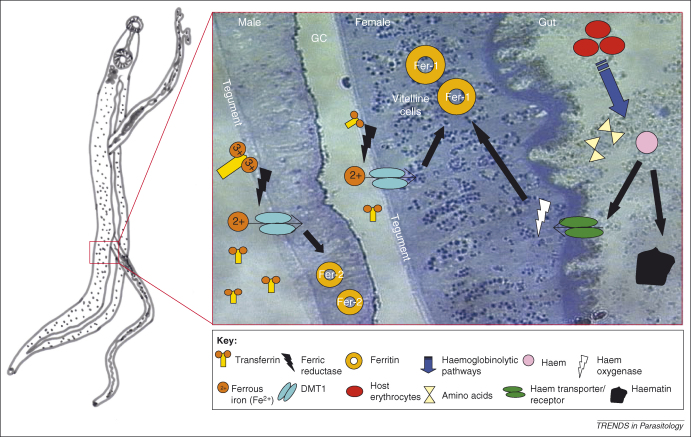
Pumping iron: hypothesized iron uptake mechanisms in schistosomes. Iron uptake at the schistosome tegument is proposed to occur via non-specific binding of the host iron-carrier protein, transferrin (Tf). Ferric (Fe^3+^) iron is cleaved from Tf and reduced to its ferrous (Fe^2+^) form by a ferric reductase. Ferrous iron is then transported by a divalent metal transporter (DMT1). The second hypothesized mode of iron acquisition uses haem. Haem is obtained as a by-product of blood-feeding from the breakdown of host erythrocytes by a haemoglobinolytic pathway. The resulting products from this are amino acids for nutrition, and haem. Hypothesized haem uptake is via a haem transporter in the gastrodermis. Haem is then catabolized by haem oxygenase to release the iron. Excess haem is sequestered in haematin and egested from the gut. Iron taken up by the helminth is stored in ferritin (Fer); Fer-1 in the vitelline cells of females and Fer-2 in general somatic tissues. Abbreviations: GC, gynecophoric canal.

Tf and non-Tf-bound iron (NTBI) stimulate the growth of schistosomula *in vitro* [4]. The stimulatory effects of NTBI can be reversed in the presence of the iron chelator, desferroxamine. Schistosomes, therefore, might acquire iron through Tf-dependent and Tf-independent mechanisms (Figure 1). Tf binding by the tegument is non-saturable and non-specific [4], precluding the action of specific Tf-receptors, in contrast to mammalian cells and trypanosomes. *S. mansoni* expresses two isoforms of a divalent metal transporter (DMT) with significant sequence similarity to the mammalian ferrous iron-uptake protein, DMT1 (also known as Nramp2) [33]. Notably, schistosome DMT1 has been localized to the tegument and not the gastrodermis. This localization pattern complements *in vitro* studies of iron uptake in schistosomes conducted by Clemens and Basch, which suggested that iron uptake is surface-mediated and most probably from iron transporters, such as Tf, which are abundant in host serum [4]. Despite their high sequence identity to mammalian proteins, neither the schistosome ferritins nor the DMT1 sequences possess the regions associated with post-transcriptional iron regulation that are found in the homologous mammalian sequences [33,34]. Because DMT transports Fe^2+^, which is relatively insoluble at physiological pH [7], it is probable that ferric reductase is required for iron uptake, but none has been identified.

Renewed interest in haem acquisition in metazoan parasites raises the question whether schistosomes can use host haem for synthesis of haemoproteins or as a source of iron. The gastrodermis is enriched in haem by virtue of haemoglobinolytic pathways [5]. It was thought that schistosomes, like other human parasites, have no capacity to digest haem, voiding it from the gut as haematin by regurgitation. There is no *in silico* evidence that haem is catabolized to release iron, because there are no ESTs representing HO in the published schistosome EST databases [28,29]. One research group, however, has described HO activity for *Schistosoma japonicum* [35], but this requires confirmation. Given the accumulating data on haem-dependent iron uptake in metazoans [22,25], and the reasonable hypothesis that the excess haem in the schistosome gut could act as a source of iron, a search for haem utilization mechanisms is warranted.

## Therapies targeting iron transport

It is clear that iron is essential for growth and maintenance of schistosomes. Iron-uptake transporters and receptors are implicated in pathogen virulence and immunogenicity [36,37], and are generally surface located, making them favourable drug or vaccine targets. Recently, bacterial iron transporters of the outer membrane of pathogenic bacteria have been tested as potential vaccine targets with promising results [38–41]. Vaccine trials of recombinant *S. japonicum* Fer-1 in experimental schistosomiasis [42] produced only moderate protection, as expected for an intracellular protein contained within organelles. The DMTs identified in *S. mansoni* show significant overall homology to mammalian DMTs [33]. However, there are regions within the sequence with limited sequence identity and these could be targeted for vaccine development. The evidence that iron has an integral role in egg shell formation [32] means that vaccination against iron homeostasis targets could disrupt the formation of eggs, as well as the pathology and morbidity associated with egg deposition. Because adult worms alone cause no pathology and do not replicate within their mammalian hosts, targeting egg production is a desirable approach for vaccine development [43].

Another strategy for parasite control might include the use of chemotherapeutics targeted at iron uptake and regulation. The artemisinin drug family has shown to be effective against both schistosomiasis and malaria [44,45]. Although the mode of action of this group of drugs is still under investigation, there is evidence that the antiparasitic activity is iron-dependent [46–48]. The use of iron chelators is best documented in the treatment of malaria, but they have been proposed as potential chemotherapeutic agents against other parasitic diseases [49]. *In vitro*, iron chelators halt the growth of schistosomes and protozoan parasites [4,17,50].

## Concluding remarks

Although the amount of data on iron assimilation in schistosomes is growing, there remain significant gaps and inconsistencies in our knowledge (Box 2). In addition, there is no information on iron uptake and metabolism in other parasitic helminths. It is clear that iron uptake and metabolism in schistosomes represent novel areas for study. The varied nature of iron-uptake mechanisms provides numerous putative targets against which novel therapies could be directed. Elucidating how iron homeostasis and other metabolic processes differ from mammalian host cells is not only important for the development of new control strategies, but will also expand our knowledge of parasite biology.

## Figures and Tables

**Table 1:** Summary of iron uptake mechanisms in prokaryotesa,b

Protein	Mechanism and target iron source	Organism
Siderophores (e.g. coprogen, ferrichrome, enterobactin and rhodotorulic acid)	Low molecular mass iron chelators synthesized and secreted by bacteria to bind ferric iron.	Gram-negative bacteria. *Escherichia coli* is the model.
FepA, FecA and FhuA	Outer membrane siderophore receptors. Transport through the outer membrane is mediated by an energy transducing TonB-ExbB-ExbD protein complex.	*E. coli* and other Gram-negative bacteria
FhuD, FepD, FepG	Transport of siderophores across the periplasm and the cytoplasmic membrane; also uses ABC permeases to facilitate uptake.	*E. coli* and other Gram-negative bacteria.
FeoA, FeoB	Ferrous iron transporters. Important during low oxygen conditions when ferrous iron is more predominant than ferric iron. Although reductase activity facilitates this action, no specific proteins have been identified.	*E. coli*, *Salmonella*, *Helicobacter pylori*
SfuABC, SitABCD, FbpABC	Metal-type ABC transporters. Transport ferrous iron.	*Serratia marcescens*, *Salmonella typhimurium*, *Neisseria gonorrhoeae*
Tbp1, Tbp2, Lbp1, Lbp2	Outer membrane receptors for host Tf and lactoferrin.	*Pasteurellaceae*, *Neisseriaceae*, *Haemophilus* spp.
IsdC, DppBCDF, HbpA	Haem iron transporters. Use haem, haemoglobin or the haemopexin complex. Gram-negative bacteria require TonB protein complex for transport and ABC permeases.	*Bacillus anthracis*, *E. coli*, *Haemophilus influenzae*
Fur	Iron regulator. Controls the expression of iron uptake proteins post-transcriptionally in response to iron availability.	Model organism is *E. coli. Pseudomonas aeruginosa*, *Bacillus subtilis*

a Taken from [12].

b Abbreviations: Fep, ferric enterobactin protein; Fec, ferric citrate binding; Fhu, ferrihydroxamate binding; Feo, Fe-oxidising protein; Sfu, *Serratia* ferrous uptake protein; Sit, *Samonella* iron transporter; Fbp, ferric-binding protein; Tbp, Tf-binding protein; Lbp, lactoferrin-binding protein; Isd, iron-regulated surface determinant; Dpp, dipeptide permease; Hbp, haem-binding protein; Fur, ferric uptake regulator protein; ABC, ATP-binding cassette.

**Table 2:** Summary of iron sources and strategies of uptake in parasitic protozoa^a^

Organism	Iron source(s)	Mechanism
*Trypanosoma brucei*	Tf	A specific receptor-mediated uptake. The receptor is a 50–60 kDa heterodimer, and its monomers are encoded by two homologous genes: ESAG6 and ESAG7. This complex binds host Tf and endocytoses into the flagellar pocket for processing [19,51].
*Trichomonas vaginalis*	Lf and Hb	Lf uptake occurs via a specific non-saturable 136 kDa receptor [17]. Hb is utilized as an iron source *in vitro*. Non-saturable binding of Hb indicates possible receptor [17].
*Leishmania chagasi*	Tf and Hb	A Tf receptor was initially proposed [52], however, it has since been found to be non-specific [17]. An uncharacterized ferrous iron transporter might act in tandem with a reductase to facilitate uptake from Tf [18]. Hb can promote growth *in vitro*, but there is no identified uptake mechanism [17].
*L. amazonensis*	?	LIT1 facilitates ferrous iron uptake in amastigotes. The biological iron source is not confirmed [20]. Use of Tf or Hb would require cleavage from the protein and reduction to the ferrous form.
*Plasmodium* spp*.*	?	Rodriguez proposed that *Plasmodium* induces uptake of Tf receptor across the erythrocyte plasma membrane [53] but numerous groups have found that *Plasmodium* cannot access Tf-bound iron [17]. Haem-bound iron is also ruled improbable, with iron from the cytosolic pool in erythrocytes the most probable iron source [2].

a Abbreviations: Tf, transferrin; Lf, lactoferrin; Hb, haemoglobin.; ESAG, expression site-associated genes; LIT1. *Leishmania* iron transporter 1.

## Box 1 Potential iron sources from the host available for scavenging by pathogens

**Transferrin (Tf)** – a glycoprotein in blood that binds and transports two ferric ions with high affinity.

**Non-Tf-bound iron (NTBI)** – as the name suggests, this includes all forms of iron not bound in Tf, and can include iron that is weakly complexed to molecules such as albumin, citrate, amino acids and sugars.

**Haemoglobin** – an iron-containing protein pigment of red blood cells, functioning primarily in the transport of oxygen from the lungs to the body tissues.

**Haptoglobin/haemoglobin complexes** – a protein in the blood that binds free haemoglobin released from erythrocytes with high affinity and thereby inhibits its oxidative activity.

**Haemopexin/haem complexes** – a serum glycoprotein that binds haem, and transports it to the liver for breakdown and iron recovery.

**Ferritin** – a globular protein complex consisting of 24 protein subunits that is the main intracellular iron-storage protein.

**Lactoferrin** – belongs to the Tf family of proteins, and is found in milk, mucosal surfaces and secretions (such as tears and saliva).

**Myoglobin** – a monomeric haem protein found mainly in muscle tissue where it serves as an intracellular storage site for oxygen.

## Box 2 Outstanding questions concerning schistosome iron uptake

Do schistosomes acquire iron directly from host Tf?

What is the identity of the ferric reductase(s) that provides ferrous iron to DMT1?

Is haemoglobin digestion the primary source of iron for schistosomes: if so, by what mechanism?

Do schistosomes have post-transcriptional regulation of iron uptake similar to that seen in prokaryotes and mammalian cells?
